# Prevalence of premenstrual syndrome and its association with psychosocial and lifestyle variables: a cross-sectional study from Palestine

**DOI:** 10.1186/s12905-021-01374-6

**Published:** 2021-06-05

**Authors:** Reem Abu Alwafa, Manal Badrasawi, Raheeq Haj Hamad

**Affiliations:** 1 Nutrition and Food Technology Department, Faculty of Agriculture and Veterinary Medicine, An-Najah National University, Nablus, Palestine; 2 Nursing and Midwifery Department, Faculty of Medicine and Health Sciences, An-Najah National University, Nablus, Palestine

**Keywords:** Premenstrual syndrome, Menstruation disturbances, Dietary habits, Mental health

## Abstract

**Background:**

Premenstrual Syndrome (PMS) is a very common problem with symptoms that can negatively affect normal daily life. This cross-sectional study aimed to investigate the prevalence of PMS symptoms and their relationship with psychosocial status and lifestyle of female students at An-Najah National University in Palestine. A sample of 398 female students was randomly selected to participate in the study. Arabic Premenstrual Scale (A-PMS) was used for PMS assessment. Psychosocial variables were determined using the DASS-21 Arabic version, and dietary habits were measured using a 24 item self-reported questionnaire. Data was analyzed by one-way ANOVA and Chi-square tests using SPSS software version 23.

**Results:**

The 398 participants (100%) suffered from some kind of PMS symptoms; 398 (100%) had physical symptoms, 397 (99.7%) had psychological symptoms, and 339 (85.2%) had behavioral PMS symptoms. All PMS symptoms were significantly associated with student psychosocial status (*p* < 0.01). Preferring a certain type of food during menstruation was significantly related to psychological PMS symptoms (*p* < 0.001), and physical symptoms (*p* < 0.01). Following a diet was significantly related to physical symptoms (*p* < 0.05) and behavioral symptoms (*p* < 0.001). Moreover, drinking herbal tea was significantly related to physical symptoms (*p* < 0.001) and behavioral symptoms (*p* < 0.05).

**Conclusion:**

The findings of the study revealed a relatively high prevalence of PMS syndrome with a significant relationship with dietary habits and psychosocial status.

## Background

Menstruation is a normal part of every woman’s life and it is necessary for the uterine lining renewal to prepare it for pregnancy. Premenstrual syndrome (PMS) is a menstrual disorder that can be described as a group of physical, behavioral, and emotional symptoms that occurs during the last week of luteal phase, which is usually the week before the mensuration. Typically, symptoms start after the 13th day of the menstrual cycle. PMS symptoms occur at any time during reproductive years. PMS can disrupt the normal life of women for days [1]. Frank clinically described PMS as “Premenstrual Tension” for the first time in 1931. In 1953, Greene and Dalton used the term “Premenstrual Syndrome” to show that it has more symptoms than just emotional tension [2].

The reported physical and psychological symptoms occurring pre-menstrually are more than 100 symptoms [3]. Common PMS symptoms include: mood swings, depression, irritability, abdominal cramps, headache, generalized pains, abdominal bloating, breast swollen and tenderness, and appetite changes. Many theories have been developed regarding the etiology of PMS. However, most of these theories are not scientifically based or proven yet [4]. It was reported that psychosocial [5] and socio-demographic factors can affect PMS. BMI was also found to influence the presence of PMS [6] while another study showed no relationship between women BMI and PMS [7].

PMS is very common and it affects millions of young women [4]. Epidemiological data shows that 75% of women in reproductive age suffer from some PMS symptoms, while 3% to 8% reported extremely severe PMS symptoms [3]. A study on PMS prevalence among different countries worldwide found that 47.8% (95% CI: 32.6–62.9) of women have PMS [8]. Other several studies on the PMS prevalence in Middle Eastern countries had been conducted in the past 20 years. For instance, PMS percentages among university students were 71.9% in Palestine [9], 92.3% in Jordan [10], 80.2% in Egypt [11], and 63% in Lebanon [12].

PMS diagnosis depends on PMS symptoms and relationship with the luteal phase because there is no objective chemical biomarker of PMS. In the 1980s, the US National Institutes of Health proposed a PMS diagnosis criteria based on the Diagnostic and Statistical Manual III (DSM III) used to identify premenstrual dysphoric disorder (PMDD) (Table 1). However, this tool could not fully diagnose women with severe clinical PMS symptoms. Therefore, it was modified to define moderate to severe PMS by the presence of at least one physical or psychological symptom during the luteal phase that results in significant impairment [13]. The DMS III criteria had been revised three times before the latest version, DMS-5 was published in 2013 [14].

**Table 1:** Research criteria for PMDD (DSM-IV-TR) [17]

Criteria for Premenstrual Dysphoric Disorder (PMDD, modified from the Diagnostic and Statistical Manual of Mental Disorders 4th edition)
A. Symptoms must occur during the week before menses and remit a few days after onset of menses. Five of the following symptoms must be present and at least one must be (1), (2), (3), or (4)
1. Depressed mood or dysphoria
2. Anxiety or tension
3. Affective lability
4. Irritability
5. Decreased interest in usual activities
6. Concentration difficulties
7. Marked lack of energy
8. Marked change in appetite, overeating, or food cravings
9. Hypersomnia or insomnia
10. Feeling overwhelmed
11. Other physical symptoms, e.g., breast tenderness, bloating
B. Symptoms must interfere with work, school, usual activities or relationships
C. Symptoms must not merely be an exacerbation of another disorder
D. Criteria A, B, and C must be confirmed by prospective daily ratings for at least 2 consecutive symptomatic menstrual cycles

Most women can manage their PMS symptoms through conservative treatments and changes in lifestyle such as dietary modifications, stress management, daily charting of symptoms, exercising, relaxation, education [3], and sleep hygiene [15]. It is recommended to reduce or eliminate the intake of salt, caffeine, chocolate, tobacco, and alcohol, consume small frequent meals rich in complex carbohydrates, and take vitamins and minerals moderately [3]. Herbs are commonly used for treating PMS symptoms [16].

Literature on PMS shows that this problem overlooked in developing countries, including Palestine (study setting). The unstable and challenging economic, political, and social circumstances in Palestine have a huge impact on the psychosocial status of women, which in turn can affect their health. Their health condition might become worse at the time of menstrual. There is an urgent need to collect data the PMS prevalence among young women in Palestine and find its effect on their health. Such data shall be useful to prepare and implement supportive and educational programs for women with PMS by, universities, community centers and others.. Hence, this study tries to fill in this gap by investigating the PMS prevalence among Palestinian university students and its relationship with depression, anxiety, stress, and dietary lifestyle.

## Materials and methods

### Study design

This cross-sectional study took place in January and February 2019 at An-Najah National University, which is the largest university in Palestine with students from all over the country. The university has three main campuses, two in Nablus city and one in Tulkarem city. A total of 433 female students from different faculties in the three university campuses located was randomly invited to join the study and sign the consent form. A definite number of students from each campus were invited to join the study. The sampling was done using stratified random sampling. The collected data include students’ scoio-demographics, medical history, life style, dietary behaviors, psychosocial and premenstrual syndrome. The response rate was 91.9% as 35 participants were excluded from the final analysis due to missing data. Inclusion criteria were female students at An-Najah National University who agreed to get involved in the study. Those who were pregnant or disagreed to participate where excluded from the study. The researchers verbally informed all participants about the study objectives and provided them with written consents. Participants who signed the contest were included in this study.

### Sample size

The sample size was determined using a single proportion for a finite population. Sample size calculations using G power software with an alpha of 0.05 (two-sided) and 80% power, indicated that a minimum of 200 participants was needed to determine a prevalence of PMS. To determine the association between PMS and dietary habits, psychosocial factors, the sample size was recalculated with mean difference test, 5% level of significance, (80%) power, giving a sample size of 380 participants.

### Collected data and study instruments

In this study, female university students filled a self-administered structured questionnaire that included personal information and dietary habits questions, Depression, Anxiety, and Stress Scale (DASS-21) questionnaire [18], and the validated Arabic Premenstrual Syndrome Scale (A-PMS) [19].

The questionnaire included 20 personal information questions on Scio-demographic characteristics (age, academic year, faculty, living place, nature of living, marital status, monthly income, work, and university fee), smoking, physical activity (working out at the gym, working out at home, walking, work required physical activity), and medical history (chronic diseases, medications, surgery, screen, and sleeping time).

For dietary lifestyle assessment, height was self-reported and weight measurement was done by a calibrated electronic body weight scale. Body mass index (BMI) of participants was calculated by dividing body weight (kg) to height (m) square (kg/m^2^). In addition, a selected 24-item questionnaire on daily dietary habits based on a validated food frequency questionnaire from a previous study on a Palestinian sample was used [20]. Items included: eating 3 meals, eating breakfast, type of bread, eating fruits, eating vegetables, drinking milk, drinking caffeinated beverages, drinking sweetened juice, eating sweets, eating nuts, eating chips, taking dietary supplements, following a diet, satisfaction with dietary habits, body weight, and body shape, and source of nutrition information. Participants were also asked about their dietary habits during menstruation. The habits included: drinking herbal tea, type of herbal tea, food preferences and types,, and food avoidance and types. Additionally, they were asked if their menstrual cycle is regular.

The Arabic validated version of DASS-21 questionnaire included 21 questions to evaluate psychosocial status [18]. In this tool, the participants should answer the questions according to their current situation (during the past week) using 4-point Likert scale (1 = never, 2 = sometimes, 3 = often, and 4 = always) and each answer has a given score. The severity of depression, anxiety, and stress was calculated by the sum of their’ answers scores multiplied by 2 [21].

A-PMS diagnostic tool developed and validated by Algahtani and Jahrami (2014) was used for assessing PMS symptoms prevalence and severity. This tool included 23 questions on PMS symptoms categorized into psychological (depressed mood, hopelessness, feeling guilty, anxiety/ worry, affective labiality, increased sensitivity toward others, feeling angry, easily irritated/ agitated, lack of interest, difficulty in concentrating, loss of control, and feeling overwhelmed); physical (lethargy/ fatigue/ decreased energy, increased appetite, craving certain foods, hypersomnia, insomnia, breast tenderness, breast engorgement or weight gain, headache, muscle, joint, abdominal, and back pain, and acne); and behavioral (symptoms interfering with relationships, work or school, or daily routine). Each question has a 5-point Likert scale (never, sometimes, often, always and severely) and each answer has a given score. PMS symptoms categories’ severity among the sample was calculated by the mean of each category’s questions score [19]. Both DASS-21 and A-PMS questionnaires were interview based, where participants answered questions verbally and the researcher recorded it.

### Validity and reliability

The validity of the Arabic versions of DASS-21 and A-PMS questionnaires were verified and established in previous studies indicating their appropriateness for Arab samples [18, 19]. In this study, the internal consistency reliability using Cronbach’s Alpha was used to examine the reliability of the two questionnaires (i.e. DASS-21 and A-PMS Arabic versions). The Cronbach’s values were very high for both questionnaires, 0.913 and 0.910 respectively.

### Statistical analysis

Data were coded and analyzed using the Statistical Package for Social Sciences (SPSS) software version 23. Continuous and categorical variables were analyzed by frequency and descriptive analyses. One-way ANOVA and Chi-square tests were used to evaluate the association between Socio-demographic characteristics, dietary habits variables, and PMS symptoms. Univariate and multivariate simple linear regression analysis tests were done for investigating the relationship between PMS symptoms and psychosocial status and physical activity hours. Pearson correlation test and multiple linear regression test were conducted to evaluate the relationship between PMS symptoms and BMI and psychosocial status variables.

## Results

### Socio-demographic characteristics

Table 2 details that the study participant come from various faculties with majority from Medicine and Heath Sciences, 170 (43.5%); first and second years, 127 (31.9%) and 116 (29.1); village 212 (53.6) and city 183 (46.3) and others.

**Table 2:** Participants’ socio-demographic characteristics

Variable	Total (N = 289)	
	Number (N)	Percentage (%)
*Faculty*		
Medicine & health sciences	170	43.5
Engineering	30	7.7
Applied science	4	1
Economy	39	10
Human science	86	22
Agriculture	48	12.3
Law	5	1.3
Public Relations	3	0.8
Art	2	0.5
Information technology & computer engineering	1	0.3
Physical education	3	0.8
*Academic year*		
1st	127	31.9
2nd	116	29.1
3rd	78	16.9
4th	54	13.6
5th	19	4.8
Master	4	1
*Area of living*		
City	183	46.3
Village and Camp	212	53.6
*Type of housing*		
With family	326	81.9
Student housing	70	17.6
With relatives	2	0.5
*Martial status*		
Single	373	94
Married	16	4
Other	8	2
*Family income*		
< 1500 NIS	23	5.9
1500 – 3000 NIS	103	26.5
3000 – 5000 NIS	142	36.6
More than 5000 NIS	120	30.9
*Working status*		
Working regularly	6	1.5
Working unregularly	27	6.8
Not working	363	91.7
*Study funding*		
Parents	369	92.7
Scholarships	23	5.8
Other	4	1

### Lifestyle and medical history

Most of the participants 340 (86.1%) are non-smokers. Additionally most of them 382 (97.7%) did not suffer from a chronic disease. Only 14 (3.5%) go to the gym regularly and 63 (15.9%) go unregularly. More details on participants’ lifestyle are shown in Table 3.

**Table 3:** Medical history and lifestyle of participants

Variable	Total (N = 389)	
	Number (N)	Percentage (%)
*Smoking*		
Non-smoker	340	86.1
Irregular smoker	39	9.9
Regular smoker	16	4.1
*Reporter type of smoking*		
Cigarette	8	2
Pipe (shisha)	52	13.1
*Chronic disease*		
Yes	9	2.3
No	381	97.7
*Use of medications*		
Yes	6	1.5
No	391	98.5
*Medical surgery*		
Yes	68	17.3
No	325	82.7
*Regular menstruation*		
Yes	297	75.4
No	97	24.6
*Workout at gym*		
Yes	14	3.5
Sometimes	63	15.9
No	320	80.6
*Workout at home*		
Yes	28	7.1
Sometimes	198	50.1
No	169	42.8
*Walk*		
Yes	194	48.9
Sometimes	163	41.1
No	40	10.1
*Work requires physical activity*		
Yes	103	26.2
Sometimes	158	40.2
No	132	33.6

	Mean ± SD	Range
Screen time (hours/day)	5.38** ± **3.07	(1–20)
Sleeping (hours/day)	7.53** ± **1.97	(2–17)
Workout at gym (hours/week)	2.86** ± **2.28	(0.25–14)
Workout at home (hours/week)	1.52 ± 1.47	(0.08–7)
Walking time (hours/week)	3.83 ± 4.66	(0.17–49)

*SD* standard deviation

### Body weight status, daily dietary habits, and during menstruation

According to BMI categorization most of the participants 287 (72.1%) had normal weights, 46 (11.6%) were underweight, 56 (14.1%) were overweight, and 9 (2.3%) were obese. For the daily dietary habits, 154 (38.8%) had followed a diet, mainly for gaining weight, losing weight, and others. During menstruation, 228 (57.7%) drink herbal tea; 190 (48.3%) prefer a certain type of food such as sweets and citrus fruits; and 103 (26.4%) avoid a certain type of food such as salt and sugar (Table 4).

**Table 4:** Daily dietary habits of participants

Variable	Total (N = 389)	
	Number (N)	Percentage (%)
*Eat three meals*		
Yes	167	42.2
Sometimes	166	41.9
No	63	15.9
*Eat breakfast*		
Yes	159	40.4
Sometimes	148	37.6
No	87	22.1
*Type of bread*		
White	249	63.7
Whole grain	26	6.6
Both	116	29.7
*Eat fruit daily*		
Yes	99	25
Sometimes	208	52.5
No	89	22.5
*Eat vegetables*		
Yes	135	34.4
Sometimes	184	46.8
No	74	18.8
*Drink milk*		
Yes	106	26.8
Sometimes	160	40.5
No	129	32.7
*Drink caffeinated beverages*		
Yes	283	60.1
Sometimes	87	22
No	71	17.9
*Drink sweetened juice*		
Yes	47	11.9
Sometimes	181	45.9
No	166	42.1
*Eat sweets*		
Yes	121	30.7
Sometimes	173	43.9
No	100	25.4
*Eat nuts*		
Yes	26	6.6
Sometimes	204	51.6
No	165	41.8
*Eat chips*		
Yes	71	18.1
Sometimes	163	41.5
No	159	40.5
*Take dietary supplements*		
Yes	36	9
Sometimes	90	22.6
No	272	68.3
*Followed a diet*		
Yes	154	38.8
No	243	61.2
*Satisfied with dietary habits*		
Yes	60	15.2
To some extent	226	57.1
No	110	27.8
*Satisfied with body weight*		
Yes	147	36.9
To some extent	146	36.7
No	105	26.4
*Satisfied with body shape*		
Yes	186	47
To some extent	149	37.6
No	61	15.4
*Source of nutritional information*		
Dietitians	56	15.2
Healthcare workers	27	7.3
Internet	232	63
Books	17	4.6
Other	36	9.8

### Psychosocial status

DASS test revealed that more than two thirds of the participants 288 (72.4%) suffered from a certain level of depression, 303 (76.1%) suffered from anxiety, and majority 322 (80.9%) suffered from stress (Table 5).

**Table 5:** Psychosocial status of participants

Variable	Total (N = 389)	
	Number (N)	Percentage (%)
*Depression*		
Normal	110	27.6
Mild	44	11.1
Moderate	113	28.4
Severe	55	13.8
Extremely severe	76	19.1
*Anxiety*		
Normal	95	23.9
Mild	30	7.5
Moderate	72	18.1
Severe	64	16.1
Extremely severe	137	34.4
*Stress*		
Normal	76	19.1
Mild	107	26.9
Moderate	106	26.6
Severe	76	19.1
Extremely severe	33	8.3

### The prevalence of premenstrual syndrome symptoms

All participants 398 (100%) suffered from some type of PMS symptoms The most frequent PMS symptoms were lethargy/ fatigue/ decreased energy 353 (88.7%), affective labiality 352 (88.5%), depressed mood 346 (87%), difficulty in concentrating 335 (84.2%), anxiety/ worry 334 (83.9%), lack of interest 332 (83.4%), easily irritated/ agitated 329 (82.6%), and increased sensitivity toward others 322 (80.9%). Most frequent severe physical symptoms were muscle, joint, abdominal, and back pain 345 (38.2%) and lethargy/ fatigue/ decreased energy 353 (34.2%) (Table 6).

**Table 6:** Prevalence of premenstrual syndrome symptoms by the level of severity (n = 398)

Symptom	None	Mild	Moderate	Severe	Total
	N (%)	N (%)	N (%)	N (%)	N (%)
Psychological symptoms					
Depressed mood	52 (13.1)	131 (32.9)	120 (30.1)	95 (23.9)	346 (87)
Hopelessness	86 (21.6)	139 (34.9)	117 (29.4)	56 (14.1)	312 (78.4)
Feeling guilty	183 (46)	97 (24.4)	72 (18.1)	46 (11.5)	215 (54)
Anxiety/ worry	64 (16.1)	113 (28.4)	121 (30.4)	100 (25.1)	334 (83.9)
Affective labiality	46 (11.6)	99 (24.9)	117 (29.4)	136 (34.2)	352 (88.5)
Increased sensitivity toward others	76 (19.1)	114 (28.6)	103 (25.9)	105 (26.4)	322 (80.9)
Feeling angry	187 (47)	74 (18.6)	84 (12.1)	53 (13.3)	211 (53)
Easily irritated/ agitated	69 (17.3)	104 (26.1)	118 (29.6)	107 (26.9)	329 (82.6)
Lack of interest	66 (16.6)	94 (23.6)	130 (32.7)	108 (27.1)	332 (83.4)
Difficulty in concentrating	63 (15.8)	132 (33.2)	114 (28.6)	89 (22.4)	335 (84.2)
Loss of control	84 (21.1)	108 (27.1)	111 (27.9)	95 (23.9)	314 (78.9)
Feeling overwhelmed	99 (24.9)	96 (24.1)	121 (30.4)	82 (20.6)	299 (75.1)
Physical symptoms					
Lethargy/ fatigue/ decreased energy	45 (11.3)	98 (24.6)	119 (29.9)	136 (34.2)	353 (88.7)
Increased appetite	117 (29.4)	91 (22.9)	92 (23.1)	98 (24.6)	281 (70.6)
Craving certain foods	90 (22.6)	108 (27.1)	97 (24.4)	103 (25.9)	308 (77.4)
Hypersomnia	93 (23.4)	104 (26.1)	99 (24.9)	102 (25.6)	305 (76.6)
Insomnia	112 (28)	115 (28.9)	96 (24.1)	75 (19)	286 (72)
Breast tenderness	170 (42.7)	95 (23.9)	74 (18.6)	59 (14.8)	228 (57.3)
Breast engorgement or weight gain	167 (42)	80 (20.1)	91 (22.9)	60 (15.1)	231 (58.1)
Headache	97 (24.4)	115 (28.9)	102 (25.6)	84 (21.1)	301 (75.6)
Muscle, joint, abdominal, and back pain	53 (13.3)	83 (20.9)	110 (27.6)	152 (38.2)	345 (86.7)
Acne	88 (22.1)	102 (25.6)	101 (25.4)	107 (26.9)	310 (77.9)
Behavioral symptoms					
Symptoms interfering with:					
Relationships	167 (42)	107 (26.9)	73 (18.3)	51 (12.8)	231 (58)
Work or school	152 (38.2)	113 (28.4)	78 (19.6)	55 (13.8)	246 (61.8)
Daily routine	102 (25.6)	102 (25.6)	115 (29)	79 (19.8)	296 (74.4)
Cumulative psychological symptoms	1 (0.3%)	107 (26.9%)	209 (52.5%)	81 (20.4%)	397 (99.7%)
Cumulative physical symptoms	0 (0%)	116 (29.1%)	209 (52.5%)	73 (18.3%)	398 (100%)
Cumulative assessment of behavioral symptoms	59 (14.8%)	169 (42.5%)	116 (29.1%)	54 (13.6%)	339 (85.2%)
Cumulative overall PMS symptoms	0 (0%)	98 (24.6%)	232 (58.3%)	68 (17.1%)	398 (100%)

### Relationship between premenstrual syndrome symptoms & depression, anxiety and stress

Univariate analysis showed that all psychological, physical, and behavioral PMS symptoms were significantly related to depression, anxiety, and stress, *p* < 0.01, meaning the higher the severity of PMS symptoms, the higher the score of depression, anxiety, and stress. Only depression scores and PMS physical symptoms were not significantly related using the multivariate analyses, *p* > 0.05 (Table 7).

**Table 7:** Relationship between premenstrual syndrome symptoms & depression, anxiety and stress

PMS Symptoms	Factors	Univariate Analysis^a^		Multivariate Analysis^b^		
		Beta (95% CI)	*p* value	Beta (95% CI)	*p* value	R square
Psychological	Depression	.459 (.049–.072)	.000**	.139 (.003–.034)	.021*	.310
	Anxiety	.447 (.049–.073)	.000**	.133 (.003–.034)	.022*	
	Stress	.534 (.060–.082)	.000**	.350 (.030–.063)	.000**	
Physical	Depression	.240 (.017–.041)	.000**	-.077 (-.025–.006)	.245	.156
	Anxiety	.349 (.032–.055)	.000**	.208 (.010–.042)	.001*	
	Stress	.365 (.033–.056)	.000**	.280 (.017–.051)	.000**	
Behavioral	Depression	.373 (.048–.079)	.000**	.176 (.008–.052)	.007*	.178
	Anxiety	.353 (.046–.078)	.000**	.139 (.003–.046)	.027*	
	Stress	.380 (.049–.080)	.000**	.166 (.005–.052)	.020*	

^*^p < .05; **p < .01 using simple linear regression/multiple linear regression

^a^Univariate analysis using simple linear regression

^b^Multivariate analysis using multiple linear regression

### Relationship between premenstrual syndrome symptoms & socio-demographic characteristic:

No significant relationship was found between socio-demographic characteristics and PMS symptoms, *p* > 0.01.

### Relationship between premenstrual syndrome symptoms and body weight status and dietary habits

No association between BMI categories of participants and all PMS symptoms was found. Similarly, no significant relationship was found between physical activity hours and PMS symptoms except for walking hours. Multivariate analysis found that walking hours were significantly related to PMS behavioral symptoms (*p* < 0.05) (Table 7).

No significant relationship was found between psychological symptoms and dietary habits of participants. Moreover, there was a significant relationship (*p* = 0.001) between psychological symptoms and preferring some type of food during menstruation. Those who prefer some type of food were less likely to suffer from mild psychological symptoms (21.6%) than those who do not prefer some type of food (32%). In addition, participants who prefer some type of food were slightly less likely to suffer from moderate psychological symptoms (50%) than those who do not prefer some type of food (54.2%). On the other hand, the percentage of participants who prefer some type of food during menstruation and suffered from sever psychological symptoms (28.4%) was higher than those who do not prefer some type of food and severely suffered from psychological symptoms (13.3%).

Physical symptoms among participants were significantly related to following a diet (*p* < 0.05). The differences were more observed in those who mildly or severely suffered from physical symptoms. Participants who followed a diet suffered less (23.4%) from mild physical symptoms than those who did not follow a diet (32.9%). In contrast, participants who followed a diet suffered more from severe physical symptoms (25.3%) than those who did not follow a diet (14%). The satisfaction with body shape was also significantly related to physical symptoms (*p* < 0.05). The participants who were not satisfied with their body shape suffered less from moderate physical symptoms (41%) than those who were satisfied (54.8%) and satisfied to some extent (53.7%) with their body shape. However, participants who were not satisfied with their body shape suffered more from severe physical symptoms (31.1%) than those who were satisfied (14%) and satisfied to some extent (18.8%) with their body shape. Similar to psychological symptoms, preferring a certain type of food during menstruation was found to be significantly related to physical symptoms (*p* = 0.00). Participants who do not prefer a certain type of food suffered more from mild physical symptoms (39.9%) than those who prefer a certain type of food (17.9%). On the other hand, participants who prefer a certain type of food during menstruation were more likely to suffer from severe physical symptoms (26.3%) than those who do not prefer a certain type of food (11.3%). Moreover, those who prefer a certain type of food were more likely to suffer from moderate physical symptoms (55.8%) than those who do not prefer a certain type of food (48.8%). Additionally, there was a significant relationship between drinking herbal tea during menstruation and physical symptoms (*p* = 0.001). Participants who do not drink herbal tea suffered more from mild physical symptoms (38.3%) than those who drink it (22.4%). While who drink herbal tea suffered more from severe physical symptoms (22.8%) than those who do not drink it (12.6%), as well as from moderate physical symptoms (54.8%) than participants who do not drink it (49.1%).

Similar to physical symptoms, a significant relationship was found between following a diet and behavioral symptoms (*p* = 0.001). 17.3% of participants who did not follow a diet and 11% of participants who follow a diet did not suffer from behavioral symptoms. Those who did not follow a diet were more likely to have mild behavioral symptoms (46.9%) than those who followed a diet (35.1%). While participants who followed a diet were more likely to have moderate behavioral symptoms (33.1%) than those who did not follow a diet (26.7%). Additionally, participants who followed a diet were more likely to have severe behavioral symptoms (20.8%) than those who did not follow a diet (9.1%). Same as physical symptoms, drinking herbal tea during menstruation was significantly related to behavioral symptoms (*p* < 0.05). 19.2% of participants who do not drink herbal tea and 11.8% of those who drink it did not have any behavioral symptoms. The percentage of participants who drink herbal tea and had mild behavioral symptoms was slightly higher (44.3%) than those who do not drink herbal tea (40.1%). Likewise, the percentage of participants who drink herbal tea and suffered from severe behavioral symptoms was higher (17.1%) than those who do not drink herbal tea (9%). The percentage of participants who drink herbal tea and had moderate behavioral symptoms was lower (26.8%) than those who do not drink herbal tea (31.7%) (Table 8).

**Table 8:** Relationship between PMS and participants’ physical activity

PMS Symptoms	Factors	Univariate Analysis^a^		Multivariate Analysis^b^		
		Beta (95% CI)	*p* value	Beta (95% CI)	*p* value	R square
Psychological	Workout at gym	.018 (−.073 to .085)	.884	.144 (−.063 to .151)	.411	.219
	Workout at home	.027 (−.058 to .085)	.713	−.282 (−.254 to .024)	.102	
	Walking	.054 (−.009 to .024)	.350	.328 (−.002 to .085)	.063	
Physical	Workout at gym	−.153 (−.115 to .026)	.209	−.070 (−.125 to .085)	.699	.168
	Workout at home	.092 (−.024 to .106)	.216	−.225 (−.224 to .049)	.202	
	Walking	.030 (−.011 to .020)	.606	.340 (−.002 to .084)	.062	
Behavioral	Workout at gym	.100 (−.055 to .132)	.416	.188 (−.056 to .189)	.275	.243
	Workout at home	.114 (−.019 to .160)	.124	.016 (−.152 to .167)	.922	
	Walking	−.008 (−.023 to .020)	.891	.411 (.010 to .111)	.020*	

^*^p < .05; **p < .01 using simple linear regression/multiple linear regression

^a^ Univariate analysis using simple linear regression

^b^Multivariate analysis using multiple linear regression

#### Correlation and regression

Correlation test was conducted between PMS, physical activity, BMI, and psychosocial status variables. It was found that PMS was significantly correlated with BMI and psychosocial status of the study sample (Table 9). A multiple linear regression was done to evaluate the prediction of PMS score based on BMI and psychosocial status variables. A significant regression equation was found (F(4, 393) = 44.390, *p* < 0.000), with an R^2^ = 0.311.

**Table 9:** Correlation coefficients of PMS

	BMI	Depression	Anxiety	Stress
PMS	.126*	.419**	.460**	.510**

^*^ Correlation was significant at the 0.05 (2-tailed)

^**^ Correlation was significant at the 0.01 (2-tailed)

## Discussion

This study successfully determined the prevalence of PMS and its relationship with psychosocial status and lifestyle among a representative sample of Palestinian university students at the largest university in Palestine. All of the study participants 398 (100%) suffered from some kind of PMS symptoms. Among them 232 (58.3%) and 68 (17.1%) had moderate and severe PMS symptoms respectively. These results are higher than those obtained from a Palestinian study on the prevalence of PMS among university students (71.9%) [9]. This difference might be resulted from the different diagnostic tool used in the study and the fact that the selected sample included students from Medicine and Health Sciences College only. A study conducted at a university in the UAE used the same tool used in the current study showed that all participants (100%) had PMS symptoms, 55% had moderate PMS symptoms and 8% had severe symptoms [7].

### PMS psychological symptoms

Prevalence of PMS psychological symptoms among our sample were 99.7%. 52.5% had moderate psychological symptoms while 20.4% had severe psychological symptoms. The same high percentages (99.7%) was reported among university students in UAE where 47.3% of them had moderate psychological symptoms and 13% suffered from severe psychological symptoms [7]. A close percentage of psychological symptoms prevalence among Saudi women was found (97.2%), 50.7% had moderate psychological symptoms and 11.2% had severe psychological symptoms. However; the age range of their sample was wider than ours [19]. The higher prevalence of severe psychological symptoms in our sample might be caused by the hard circumstances in Palestine.

### PMS physical symptoms

Physical PMS symptoms were the most common symptoms among our sample. All participants suffered from a certain level of physical symptoms (100%). More than half of them had moderate physical symptoms (52.5%), while 18.3% had severe physical symptoms. Similarly, university students in UAE had a high prevalence of physical PMS symptoms (99.3%), while 57.7% suffered from moderate physical symptoms and 8.7% suffered from severe moderate physical symptoms [7]. A slightly lower prevalence of physical PMS symptoms was found in Saudi women (97.9%), and 45% suffered from physical symptoms moderately, while 10.1% suffered severely from physical symptoms [19]. Similar to psychological symptoms, our sample had higher prevalence of severe physical symptoms.

### PMS behavioral symptoms

The normal life of women with PMS can be negatively altered for days [1]. In our study, PMS symptoms affected 85.2% of participants’ behaviors, 29.1% had moderate behavioral symptoms and 13.6% had severe behavioral symptoms. The effect of PMS on behavior was represented in the significant (*p* < 0.05) correlation between PMS and the absence from university in a Palestinian previous study. It was found that 48.1% of students with mild PMS symptoms and 51.9% with moderate or severe symptoms often or always missed classes because of PMS symptoms [9]. 77.7% university students in UAE were found to have behavioral symptoms, and 20% and only 2% had moderate and severe behavioral symptoms, respectively [7]. A lower prevalence of behavioral symptoms was shown among Saudi women with PMS, 67.2%. and only 18.3% and 3.2% n suffered from moderate and severe behavioral symptoms [19]. The different age group might be the reason behind the lower influence of PMS on behavior found in the Saudi study. University students might have more difficulty in performing their duties while suffering from some kind of PMS symptoms.

### Most common PMS symptoms

The current study reported the most common PMS symptoms that are lethargy/ fatigue/ decreased energy (88.7%), affective labiality (88.5%), hopelessness (87.4%), and depressed mood (87%). Moreover, the most frequent severe physical symptoms were muscle, joint, abdominal, and back pain (38.2%) and lethargy/ fatigue/ decreased energy (34.2%). Similarly, lethargy (89.9%), depressed mood (88.5%), muscle, joint, and back pain (86.7%) were the key PMS symptoms in Saudi women in addition to feeling of anger (89.6%) [19]. The most prevalent PMS syndrome among university students in UAE also included depressed mood (95%), lethargy/ fatigue/ decreased energy (92%), muscle, joint, abdominal, and back pain (89.3%), and feeling of anger (85.7%). Similar to our study, muscle, joint, abdominal, and back pain (29.3%) was reported as the most common severe physical symptom [7]. A study done in Jordan showed that lower back pain (61.4%), abdominal cramps (52.4%), sadness and depression (45.3%) were among the most common complaints in women with PMS [6]. The lower percentages of these symptoms’ prevalence compared to our study might be due to the different diagnostic tool used as well as the wider age range of their sample.

### Factors affecting PMS

Our data indicated that all PMS symptoms were significantly related (*p* = 0.00) to the psychosocial status. Additionally, the higher the severity of PMS symptoms the higher the score of depression, anxiety, and stress. A case–control study found a significant relationship between PMS symptoms and psychosocial status measured by DASS-21, where Iranian students with PMS suffered more from depression, anxiety, and stress [5]. A significant positive correlation was found between stress level and PMS symptoms in Jordanian women with PMS [6]. No significant relationship was found between socio-demographic characteristics and PMS symptoms among our sample. Likewise, no significant relationship was found between the severity of PMS symptoms, e age and occupation of Saudi women [19]. In contrast, the age, income level, and martial status were significantly related to PMS in Jordanian women [6]. Additionally, no association between BMI categories of participants and all PMS symptoms was found in our sample. This was also observed in students with PMS in UAE [7]. However, BMI was significantly related to PMS in women in a study conducted in Jordan [6]. The variation of the results might be caused by the difference s in age groups, diagnostic tools, and countries’ conditions.

### Dietary habits and PMS

Following a diet was significantly related to physical symptoms (*p* < 0.05) and behavioral symptoms (*p* = 0.001). Following a diet was less related to having mild physical symptoms and more related to having severe physical symptoms. The percentage of participants who followed a diet and did not suffer from behavioral symptoms was less than those who did not follow a diet. Similar to physical symptoms, following a diet was less related to having mild behavioral symptoms. Moreover, following a diet was more related to having severe as well as moderate behavioral symptoms. Consuming food that is high in calorie, fat, sugar, or salt was found to be significantly related to higher physical symptoms reporting risk among students in the UAE [7]. Similarly, a Turkish study reported that students who consumed a high fat high calorie diet were significantly more likely to have PMS [22]. It is observed that the overall diet had an impact on PMS, but since we do not know what types of diet our participants follow the exact effect of their diets on PMS is not clear. Drinking different types of herbal tea is a common alternative treatment method for PMS in Asia [16]. Participants in this study drink herbal tea types such as mint, sage, and green tea during menstruation. We have found that drinking herbal tea was significantly related to physical symptoms (*p* = 0.001) and behavioral symptoms (*p* < 0.05) among our sample. Those who do not drink herbal tea suffer more from mild physical symptoms, while participants who drink herbal tea suffered more from moderate and severe physical symptoms. For behavioral symptoms, the percentage of participants who do not drink herbal tea and did not have any behavioral symptoms was higher than those who drink it. Additionally, percentage of participants who drink herbal tea and had mild or severe behavioral symptoms was higher than those who do not drink herbal tea. While the percentage of participants who drink herbal tea and had moderate behavioral symptoms was lower than those who do not drink herbal tea. Hence that drinking herbal tea is used for relieving the more intense PMS symptoms. Drinking herbal tea during menstruation was common among university students in UAE (68.3%) and it was not significantly associated with PMS psychological symptoms [7]. 86.4% of Jordanian women with moderate to severe PMS use herbs for symptoms management [23].

### Strengths and limitations

The sample of this study was representative of the population. The research tools were previously validated for Arabic culture participants. The limitations of the study are that this study included students from one university only. The study identified the relationship between PMS and psychosocial status rather than the cause-effect relationship. The overall severity of PMS according to menstrual cycle was not determined in this study, which is an important subject that should be considered in future studies.

## Conclusion and recommendations

PMS symptoms were reported in all participants. The most frequent PMS symptoms were: lethargy/ fatigue/ decreased energy, most frequent severe physical symptoms were muscle, joint, abdominal, and back pain, while the most frequent moderate symptoms included lack of interest, and for mild symptoms feeling angry and feeling guilty were the most common ones. The study found significant relationship between all PMS symptoms; psychological, physical, and behavioral with depression, anxiety, and stress. No association between BMI categories of participants and all PMS symptoms was found. Psychological symptoms did not have a relationship with both dietary habits and physical activity of participants. Physical symptoms among participants were significantly related to following a diet.

The high prevalence of PMS among university students requires an action to help and support those who suffer a lot. Raising awareness toward this syndrome is a necessary. Education programs can be implemented by various ways including traditional educational module or using innovative technological methods.

## Data Availability

The dataset used and analyzed in this study is available from corresponding uthor on reasonable request.

## Abbreviations

A-PMS: Arabic Premenstrual Scale
BMI: Body mass index
DASS-21: Depression, Anxiety, and Stress Scale
DSM III: Diagnostic and Statistical Manual III
NIS: New Israeli Shekel
PMDD: Premenstrual Dysphoric Disorder
PMS: Premenstrual syndrome
SPSS: Statistical Package for Social Sciences
